# Postbiotics: Current Trends in Food and Pharmaceutical Industry

**DOI:** 10.3390/foods11193094

**Published:** 2022-10-05

**Authors:** Priyamvada Thorakkattu, Anandu Chandra Khanashyam, Kartik Shah, Karthik Sajith Babu, Anjaly Shanker Mundanat, Aiswariya Deliephan, Gitanjali S. Deokar, Chalat Santivarangkna, Nilesh Prakash Nirmal

**Affiliations:** 1 Department of Animal Sciences and Industry, Food Science Institute, Kansas State University, Manhattan, KS 66506, USA; 2 Department of Food Science and Technology, Kasetsart University, Bangkok 10900, Thailand; 3 Sargento Foods, 305 Pine Street, Elkhart Lake, WI 53020, USA; 4 Department of Agriculture and Environmental Sciences, National Institute of Food Technology Entrepreneurship and Management (NIFTEM), Sonipat 131028, India; 5 Kraft Heinz R&D Center, 801 Waukegan Rd, Glenview, IL 60025, USA; 6 Department of Quality Assurance, MET’s Institute of Pharmacy, Bhujbal Knowledge City, Nashik 422003, India; 7 Institute of Nutrition, Mahidol University, 999 Phutthamonthon 4 Road, Salaya, Nakhon Pathom 73170, Thailand

**Keywords:** postbiotics, bioactivity, lactic acid bacteria, health-promoting, metabolites, functional foods

## Abstract

Postbiotics are non-viable bacterial products or metabolic byproducts produced by probiotic microorganisms that have biologic activity in the host. Postbiotics are functional bioactive compounds, generated in a matrix during anaerobic fermentation of organic nutrients like prebiotics, for the generation of energy in the form of adenosine triphosphate. The byproducts of this metabolic sequence are called postbiotics, these are low molecular weight soluble compounds either secreted by live microflora or released after microbial cell lysis. A few examples of widely studied postbiotics are short-chain fatty acids, microbial cell fragments, extracellular polysaccharides, cell lysates, teichoic acid, vitamins, etc. Presently, prebiotics and probiotics are the products on the market; however, postbiotics are also gaining a great deal of attention. The numerous health advantages of postbiotic components may soon lead to an increase in consumer demand for postbiotic supplements. The most recent research aspects of postbiotics in the food and pharmaceutical industries are included in this review. The review encompasses a brief introduction, classification, production technologies, characterization, biological activities, and potential applications of postbiotics.

## 1. Introduction

Recently, customers have shown increased interest in considering and buying healthier foods as they have grown much more mindful of the value of nutrition and health. Therefore, there is a rise in food products with functional claims and it is interesting to note that these functional foods have no internationally accepted definition [1]. In addition to fermented traditional foods, some of the primary categories of functional foods include nutraceuticals, probiotics, prebiotics, and synbiotics [2]. Probiotics are live microbes that provide health benefits when ingested, generally by improving or restoring the gut microflora. Prebiotics are a class of nutrients that the gut flora breaks down. Indeed, prebiotics appear to be exciting prospects for boosting human health as a replacement or in conjunction with probiotics, given their health benefits and advantages in manufacturing/storage over probiotics. Synbiotics are a combination of probiotics and prebiotics that either function separately to accomplish one or more health benefits or work together to offer a health benefit that might not be observed when administered separately. However, in recent years, new terms have been added, such as paraprobiotics (inactive cells of probiotics) and postbiotics (metabolites of probiotics), as research has revealed that dead cells, whether intact/ruptured, may also have significant human health. The health effects of the intestinal microbiomes are dependent on the viability of the microbiome and is also dependent on inanimate microbiome products, whereas postbiotics, which are derived from the probiotic cells, when received in adequate quantities, possess health benefits for the host [3,4]. Therefore, postbiotics can be defined as metabolites or components produced by microbiota that have an impact on human health. However, the health-improving properties or aspects of postbiotics and their bioactivities, are still unknown or unclear [5]. The International Scientific Association for Probiotic and Prebiotics defines postbiotics as “a preparation of inanimate microorganisms and/or their components that confers a health benefit on the host”. It is possible to alter scientific understanding and the advantages these postbiotic compounds have on one’s diet and overall health, thanks to the new technologies, methods, and applications that are currently under investigation [6,7].

Nevertheless, despite this development, there are still many unanswered problems, particularly with regard to the immunological response that postbiotics induce in the host. In general, the producer strains from bacterial and fungal species (*Lactobacillus*, *Bifidobacterium*, *Streptococcus*, *Akkermansia muciniphila*, *Saccharomyces boulardii*, *Eubacterium hallii*, *Faecalibacterium*, etc.), from which the postbiotics are recovered in situ can be found naturally in a variety of fermented foods. Since researchers have established a link between the microbiome and immunological support as well as general health over the past few years, gut health and food products helpful in gut health have attracted a lot of interest. Postbiotics are the newest addition to this conversation about gut health. When prebiotics are ingested, gastrointestinal bacteria ferment them and bioactive postbiotics are the byproducts of this fermentation process. Due to the existence of metabolic postbiotics or paraprobiotics, some advantages associated with the use of probiotic products may, therefore, be acquired. Although, there are several studies on the advantages of postbiotics and researchers are still trying to figure out how postbiotic production and the subsequent health consequences are related. Typical nutrients like vitamin B12, vitamin K, and folate, and several amino acids that can be produced by gut bacteria are examples of postbiotics. Other types include lipopolysaccharides, enzymes, short-chain fatty acids, bacterium lysates, and cell-free supernatants. Most recent studies contrast the ingestion of pre- and probiotic meals with supplemental intake. Immune function and relief from digestive issues are two areas that are promising. According to certain research, postbiotic supplementation may help with irritable bowel syndrome and inflammatory bowel disease symptoms as well as the prevention of respiratory tract infections. Although the precise processes are yet known, in vitro research has shown that postbiotics have antibacterial, anti-inflammatory, immunomodulatory, anti-proliferative, and antioxidant activities. This suggests that these bioactive substances may help to improve human health.

Additionally, the use of postbiotics as functional ingredients in foods offers several benefits during the industrial handling and commercialization of food products, including the ability to be added to some foods that are thought to be detrimental to the survival of probiotics. This helps to grow the market for functional foods. Practically speaking, because their viability is not necessary for either large-scale production or consumption, postbiotics are more stable and secure for food and pharmaceutical uses than the living bacteria they are produced from [8]. A change in processing conditions or scaling that can result in structural modifications and variations in the physiological function of postbiotics may make it difficult to produce postbiotics on a large scale in the industry. This is true even though the production of these compounds is possible at the laboratory scale or under small-scale conditions. In this review, the classification of postbiotics and their methods of action in the host are discussed, with an emphasis on how they interact with the immune system and the gut microbiota. The methods used for the characterization of postbiotics are also briefly discussed, along with their applications in the food and pharmaceutical industry.

## 2. Methods

Electronic searches of the literature, including those using the databases Google Scholar and PubMed were used for this review. A broad range of search terms were used for the purpose of the review including postbiotics, applications of postbiotics, production of postbiotics, exopolysaccharides, short chain fatty acids (SCFAs), bacterial lysates, in vitro studies on postbiotics, in vivo studies on postbiotics, characterization of postbiotics, postbiotics definition, the scope of postbiotics, infection prevention of postbiotics, etc. The search process revealed about 250 scholarly articles including research articles, reviews, books, patents, and other publicly available internet sources of which 200 were short-listed and used to prepare this manuscript. This review outlines a brief introduction, production technologies, characterization, classification, biological activities, and potential applications of postbiotics. This review was written based on the PRISMA guidelines and is correct to the best of our knowledge.

## 3. Classification of Postbiotics

**Figure 1:**
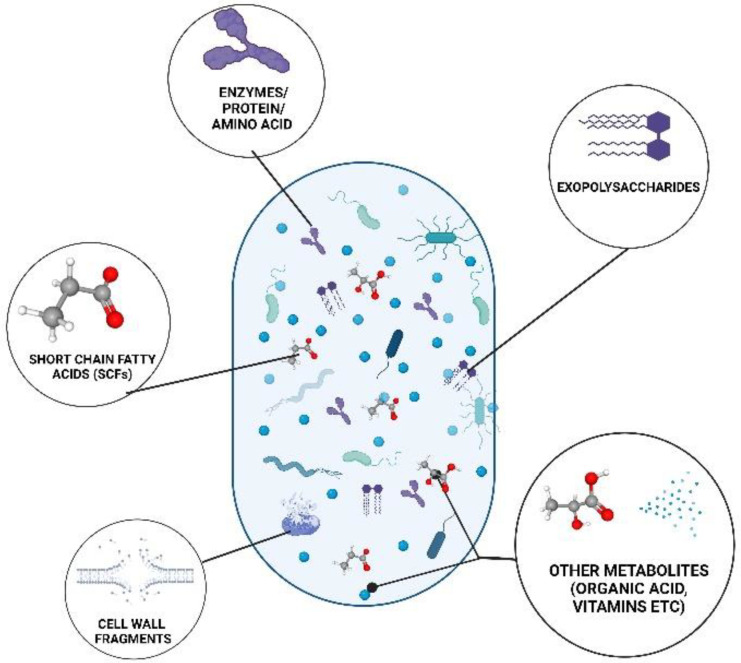
Schematic representation of postbiotic classification.

Postbiotics are classified as the metabolites generated by the microbiota, such as SCFAs, exopolysaccharides, cell wall fragments, enzymes/proteins, and other metabolites (Figure 1). Postbiotics could also be classified as structural, such as peptides, teichoic acids, and plasmalogens [9]. They could also be classified based on their elemental composition as carbohydrates (teichoic acids and galactose-rich polysaccharides), proteins (p40, p75 molecule, lactocepin), lipids (butyrate, acetate, propionate, lactate, dimethyl acetyl-derived plasmalogen), vitamins (B-group vitamins), organic acids (3-phenyllactic acid and propionic), and other complex molecules (lipoteichoic acids, peptidoglycan-derived muropeptides) [3,10]. They could also be classified by their physiological function as anti-obesogenic, antioxidant, anti-inflammatory, hypocholesterolemic, anti-hypersensitive, and anti-proliferative effects exhibiting immunomodulatory properties [11].

### 3.1. Short Chain Fatty Acids

SCFAs are an important group of metabolites that are produced by intestinal bacteria through fermentation from plant polysaccharides [12]. Prebiotics like fructooligosaccharides and inulin are fermented to produce SCFAs, particularly acetate, propionate, and butyrate, and these are present in an estimated molar ratio of 60:20:20 in the colon and feces [13]. Butyrate is one of the most important trophic sources for enterocytes as it aids in regenerating the intestinal epithelium [14]. It also has anti-inflammatory characteristics because it prevents nuclear factor-kappa B (NF-B) from becoming activated by reducing the expression of pro-inflammatory cytokines. It was also observed that significant amounts of butyrate produced by intestinal colonization with *Roseburia intestinalis* inhibit atherogenesis in a mouse model [15]. Acetate is the most abundant SCFA which is mostly produced as a fermentation product by enteric bacteria and also could be produced from formate through the Wood-Ljungdahl pathway by hydrogenotrophic bacteria such as *Acetobacterium woodii* [9]. The higher concentration of SCFAs are in the cecum; however, the SCFA concentration varies through the intestine. Another SCFA that serves as a major substrate for gluconeogenesis in the liver is propionate and, therefore, it is present only in trace amounts in the peripheral tissues [14,16]. Propionate also shows an anti-inflammatory effect (in-vivo) similar to burytate [17]. The glycolytic process produces pyruvate, which Lactobacilli can ferment to make SCFAs. They can also produce SCFAs by using the phosphoketolase pathway under heterofermenting circumstances [18]. Bifidobacteria produces acetate and lactate when carbohydrates are in excess for its growth, and when growing under carbohydrate’s limitation, it produces acetate and formate using the fermentation pathway [19]. In another study, the impact of fatty acids produced by *Lactobacillus acidophilus*, *Lactobacillus fermentum*, *Lactobacillus paracasei* ATCC 335, and *Lactobacillus brevis* opposed *Klebsiella oxytoca*. They discovered that these probiotic bacteria inhibit the growth of *Klebsiella oxytoca* by lysing the cell wall [20]. Although SCFAs can have a variety of advantageous effects on several facets of human energy metabolism, our knowledge of the underlying molecular pathways is still lacking. Since the majority of the results were obtained in rodents and cannot be directly applied to humans, this predicament is partially caused by a lack of human data. Although excessive quantities of SCFAs were discovered to have harmful effects on the epithelium in rats, it is widely recognized that SCFAs are necessary for sustaining colon epithelial health and colon health in general [21,22]. Colonic epithelial cells are expected to have anti-inflammatory effects from SCFAs, particularly butyrate. Butyrate is thought to exert its antitumor effects via blocking histone deacetylases, which control gene expression in cells. SCFA appears to have both pro- and antitumor effects. In addition to its well-known direct inhibitory effects on cell growth, SCFA may also directly affect immune cells through modification of cell signaling, epigenetic regulation, and metabolism [21].

### 3.2. Exopolysaccharides

Exopolysaccharides (EPS) are branched, repeating units of sugars or sugar derivatives that are long-chain, high-molecular-weight polymers and are produced by mostly lactic acid bacteria (LAB). EPS can be classified into two depending on the chemical composition as homopolysaccharides, which contain only one type of monosaccharide unit (examples include cellulose, levan, curdlan, pullulan, and dextran, etc.) and heteropolysaccharides which contain repeating units of several different monosaccharides (examples include xanthan, gellan, galactan, and kefiran, etc.) [23]. They wrap most bacterial envelopes and play a major role in cell adhesion and protection. EPS produced by LAB have a wide range of structural variations, and this diversity allows the polymers to have a variety of bioactivities. These biopolymers have the potential to be used in biomedical and pharmaceutical applications due to their immunomodulatory, antitumor and antimutagenicity, antioxidant, anti-inflammatory, antihypertensive activity, antibacterial and antiviral, cholesterol-lowering, and anti-gastrointestinal activity [24]. Antimicrobial and antioxidant characteristics can be found in some EPSs made by *Lactobacillus* strains isolated from fermented durian fruit. Additionally, EPSs can improve lipid metabolism by preventing the absorption of cholesterol [25]. EPS derived from *Lactococcus lactis* subsp. *lactis* showed antioxidant activity by increasing antioxidant enzymes like catalase, glutathione peroxidase, and superoxide dismutase activities and lowering lipid peroxidation levels in serum and in the livers of mice [26]. EPS extracted from *Lactobacillus reuteri* Mh-001 were found to have immunomodulatory effects [24]. EPS has many beneficial effects, including its anti-inflammatory and antioxidant characteristics which have been extensively studied; however, its underlying mechanisms are still not fully understood. The food industry currently uses EPS as emulsifying, stabilizing, and water-binding agents as well.

### 3.3. Enzymes

Enzymes can be defined as proteins that catalyze biochemical reactions. Based on their activity or function, enzymes have been categorized into six broad groups: oxidoreductases, transferases, hydrolases, lyases, isomerases, and ligases [27]. Enzymes possess a variety of functions including physiological, biochemical, and regulatory. Enzymes are primarily derived from a small group of bacterial strains mainly *Bacillus subtilis* and *Bacillus licheniformis*; fungal strains mainly *Aspergillus niger*, and *Aspergillus oryzea* industrially [28]. Two strains of *Lactobacillus fermentum* were reported to contain a significant amount of glutathione peroxidase which was later discovered to have strong in vitro antioxidant activities [29]. *Bacillus* genus has the potential to produce a high yield of proteolytic enzymes which have exceptional stability against adverse conditions such as temperature, pH, organic solvents, oxidizing compounds, and detergents [30]. Catalase from genetically modified *Lactobacillus lactis* was reported to inhibit chemically induced colon cancer in mice [31].

### 3.4. Cell Wall Fragments

A number of components of the cell wall of bacteria are immunogenic, i.e., those substances are able to produce an immune response which includes teichoic acids, lipoteichoic acids etc. [32]. Lipoteichoic acids and teichoic acids are primary constituents of the cell wall of Gram-positive bacteria and constitute about 60% of the cell wall mass in a Gram-positive bacterium [33]. Although the immunostimulatory effects of lipoteichoic acids have been demonstrated, the information regarding its activity is unclear [14]. In a study conducted by [34], the lipoteichoic acid was extracted from K8, K88, K5-5, and K55-5 strains of *Lactobacillus plantarum* and the authors also found that different lipoteichoic acid structures across the four strains of *L*. *plantarum* led to different immunological responses from immune cells [34]. Teichoic acids play a crucial role in pathophysiology and antibiotic resistance processes [35]. Both teichoic and lipoteichoic acids demonstrate a range of bioactivities including anticancer, immunomodulatory, and antioxidant properties [36]. Despite these positive benefits, lipoteichoic acid can have negative impacts on living organisms which produces an excessive inflammatory immune response; thus, further safety analysis of lipoteichoic acid is, therefore, necessary [14].

### 3.5. Cell-Free Supernatants

Cell-free supernatants (CFS) are liquids that contain the metabolites left behind from microbial growth and any unabsorbed nutrients from the growth medium. CFS which are formed when microorganisms are fermented, have anti-inflammatory, antioxidant, and antitumor properties and are also used to treat diarrhea [37]. CFS produced by LAB can have an antibacterial effect due to organic acids, proteinaceous molecules, and fatty acids. Along with other compounds, Lactic and acetic acid are primarily responsible for the antibacterial activity of CFS from LAB [38]. Different cultures or strains of microorganisms produce CFS with varying activities. CFS produced from *Lactobacillus acidophillus* and *Lactobacillus casei* were found to have antioxidant and anti-inflammatory effects [39]; *Lactobacillus* and *Bifidobacterium* were observed to have antibacterial activity by inhibiting *E*. *coli* strains [40]; yeast cultures such as *Saccharomyces bouldarii* also displayed antioxidant and anti-inflammatory activity [39], whereas *Lactobacillus rhamnosus* GG and *Lactobacillus casei* showed anticancer properties in the colon [41]. CFS, as they are produced from safe bacteria, have the potential to be used as an alternative to common antimicrobials and may be regarded as safe for human health [38] with the exception being biogenic amines and D-lactic acid [42].

### 3.6. Bacterial Lysates

Bacterial lysates are made from bacterial cells that are broken down and are aimed to stimulate the immune system to recognize and fight infections. They are obtained by the degradation of Gram-negative and Gram-positive bacteria either chemically or physically. Recently, bacterial lysates have gained a lot of attention due to their role in lowering the frequency of recurrent respiratory tract infections in children [43] and their positive effects on chronic obstructive pulmonary disease [44]. Each bacterial strain is independently produced, harvested, put through the chosen method of inactivation, and then, if needed, lyophilized. To produce a polyvalent bacterial lysate, different lysates are combined in predetermined ratios [45]. Polyvalent bacterial lysates are available commercially in the form of capsules and tablets are taken orally. Studies from the literature suggest that bacterial lysates are strong immunomodulators capable of stimulating immunoregulatory responses in mucosal tissues and antibodies against pathogens [46]. However, there is a need for more standardized protocols for the preparation of these bacterial antigens despite their significance in public health because the literature does not fully describe lysis procedures and the procedures are not publicly available to the research community [45].

### 3.7. Other Metabolites

Other metabolites produced by bacteria include vitamins, aromatic amino acids, and phenolic-derived metabolites. Vitamins cannot be produced by humans and are obtained either through diet or from microorganisms. Gut microbiomes have the ability to de novo synthesize B group vitamins including B12, B2, B6, B9, and vitamin K (also known as menaquinone) [6]. Vitamin B is observed to have anticancer properties as they play an important role in energy generation, gene regulation, and intestinal immunity modulation. Cobalamin, often known as vitamin B12 (B12), is a vital water-soluble vitamin that is found in animal products and is necessary for maintaining the health of neurons and hematopoiesis. Recently, genes encoding enzymes involved in the manufacture of cobalamin (B12) have been found in probiotics such as *L. sanfranciscensis* [47], *L. reuteri* [48], *L. rossiae* [49] and *L. fermentum* [50], which have been demonstrated to synthesize vitamin B12 and could serve as viable alternatives for industrial production. Moreover, Supplementing with *B. animalis* subsp. lactis HNO19 showed a significant improvement in the blood levels of vitamins B6 and B12 in pregnant women [51]. Vitamin K has been shown to have positive effects on bone and cardiovascular health [52]. *Eubacterium lentum*, which produces MK-6; *Lactococcus lactis ssp*. *lactis* and *spp*. *cremoris*, which mostly produces MK-8 and MK-9; and *Bacteroides fragilis*, which generates MK-10, MK-11, and MK-12 are a few menaquinone-producing bacteria [53]. Gut microbiomes are involved in the production and metabolism of aromatic amino acids that act as bioactive molecules in the brain, kidney, and cardiovascular systems [54]. Dietary polyphenols are also produced by gut microbiota [55]. Bioactive peptides derived from food proteins are shown to have immunomodulatory and anticancer properties [14].

## 4. Production Technologies for Postbiotics

Postbiotics can be found naturally in several fermented foods like yogurt, sauerkraut, pickled vegetables, and kombucha, and are produced by various bacterial and fungal species, the strains of which mainly include those of *Lactobacillus*, *Bifidobacterium*, *Streptococcus*, *Eubacterium*, *Faecalibacterium*, and *Saccharomyces* [8,10,56]. The amount of postbiotics produced during natural fermentation cannot be controlled and may be minimal or insufficient to generate a physiological response in vivo [57]. Therefore, production methodologies have been explored by researchers to produce postbiotics in a controlled and efficient manner to enable their study and use in food, pharmaceutical, and nutraceutical applications.

Postbiotics can be derived from microbial cell compounds as well as microbial action which includes the synthesis of metabolites and products from microbial activity over a nutrient matrix [8]. Production technologies of postbiotics, as reviewed by Aguilar-Toalá et al. [10], generally involve cell disruption techniques like heat [58], high pressure [59], enzymatic treatment [60], co-culturing [61], solvent extraction [62], chemical treatment (e.g., formalin) [63], and sonication [64,65,66,67,68]. For intracellular postbiotics, the bacterial membrane is disrupted by applying a combination of the above-mentioned treatments to obtain intracellular metabolites and/or cell wall components in the form of fragments [10].

Additional extraction and purification steps, such as centrifugation, dialysis, lyophilization/freeze-drying, spray drying, and column purification, are also used to assist in obtaining procedures. Tiptiri-Kourpeti et al. [65] heat-treated *Lactobacillus casei* ATCC 393 at 100 °C for 40 min and then sonicated the cells at 50 W for 10 min and centrifuged at 13,000× *g* for 40 min, to produce a postbiotic fraction of *L*. *casei* and investigated its antiproliferative effects on cancer cells. Lee et al. [58] heat-treated cultures of *Bifidobacterium bifidum* BGN4 and *Bifidobacterium* sp. CN2 at 95 °C for 30 min to study their effects on the proliferation of macrophages.

In a study by Amaretti et al. [68], cell suspensions of *Bifidobacterium*, *Lactobacillus*, *Lactococcus*, and *Streptococcus* strains were subjected to ultrasonic disruptions in five 1-min strokes at 0 °C and centrifuged to remove cell debris. The cell-free supernatants were assayed in vitro to evaluate their antioxidant potential. Choi et al. [69] prepared fractionations of heat-killed *Lactobacillus* strains by sonication in a cooled water bath at 4 °C followed by centrifugation and ultrafiltration and found that soluble polysaccharide fractions from *L*. *acidophilus* 606 may constitute a novel anticancer agent.

Dinic et al. [59] produced a bioactive lysate from *Lactobacillus fermentum* BGHV110 by pelleting (5000 rpm, 10 min) the cells, followed by high-pressure homogenization in a French press (three passages) and lyophilization to study its effects in counteracting acetaminophen-induced hepatotoxicity in HepG2 cells. Nakamura et al. [70] used lyophilized cells from *Bifidobacterium*, *Enterococcus*, *Issatchenkia*, *Lactobacillus*, *Lactococcus*, *Leuconostoc,* and *Saccharomyces* strains that were chemically treated with 0.5 M ethanolic potassium hydroxide solution followed by sonication (300 W for 2 min), heat treatment (boiling for 1 h), and solvent extraction using diethyl ether. The fragmented cells were investigated as functional postbiotics for their use in treating dyslipidemia in an obese mouse model.

Kim et al. [62] obtained the postbiotic lipoteichoic acid isolated from *L*. *plantarum* K8 by *n*-butanol extraction and studied its effectiveness in attenuating proinflammatory signaling in the gastrointestinal tract for the treatment of Crohn’s disease. Rakhuba et al. [71] extracted postbiotic glycolipids from *Lactobacillus plantarum* B-01 by applying supercritical fluid technology using CO_2_ which yielded more product (620 µg/mg of extract) than solvent extraction (55 µg/mg of extract) in lesser time.

Aguilar-Toala et al. [72] obtained postbiotics by sonicating *L*. *casei* CRL 431 for 30 min at 42 kHz and enzymatic treatment using lysozyme/mutanolysin (1 mg/ mL) at 37 °C for 150 min and assessed its bioactivity and multifunctional properties from global metabolite profiling using Raman spectroscopy. Lee et al. [60] prepared intracellular cell-free extracts from *Lactobacillus plantarum* by incubating the strains with 1 mg/mL of lysozyme at 37 °C for 30 min followed by ultrasonic disruption for five 1-min intervals and centrifugation. The cell-free extracts were evaluated for their functional property as antioxidants. Co-culturing of *Lactococcus lactis* subsp. lactis with *Saccharomyces cerevisiae* CL01 increased the production of the postbiotic nisin by 85% according to Liu et al. [73]. Ariana and Hamedi [74] also reported enhanced production of nisin by 50% by *L*. *lactis* in the presence of *Yarrowia lipolytica* in sugar beet molasses.

Vidal et al. [75] used the column purification technique by utilizing a Octyl-Sepharose^™^ CL-4B column for isolation of lipoteichoic acid from *Lactobacillus johnsonii* La1 and *Lactobacillus acidophilus* La10. Matsuguchi et al. [76] used a combination of Macro-prep High Q and Octyl-Sepharose CL-4B column for isolation of lipoteichoic acid from *L*. *casei* YIT 9029 and *L*. *fermentum* YIT 0159. Centrifugation was used in the extraction of intracellular content from various *Bifidobacterium*, *Lactobacillus*, *Lactococcus*, and *Streptococcus* strains in different studies [64,68,77] and is one of the most common extraction methodologies for postbiotics mentioned in literature. For secreted postbiotics by viable cells, the methods of recovery from supernatants consist of eliminating the viable cells from the medium by centrifugation, or in combination with microfiltration.

For food and pharmaceutical applications, it is beneficial to process postbiotic extracts into freeze-dried (lyophilization) and spray-dried forms for ease of handling and storage. Shafipour Yordshahi et al. [78] prepared a lyophilized/freeze-dried powder containing postbiotics derived from *Lactobacillus plantarum* ATCC 14917, which were incorporated in bacterial nanocellulose to develop an antimicrobial ground meat wrapping nanopaper. Puccetti et al. [79] developed spray-dried microparticles loaded with postbiotics (indole-3-aldehyde) with gastro-resistant properties for small intestine local delivery. However, these types of processing of extracted postbiotics can influence their composition, which may decrease postbiotic activity. For example, lyophilization promotes the elimination of hydrogen peroxide, which is a factor responsible for antimicrobial activity [80]. An increase in pH due to chemical treatment can also affect antimicrobial activity [81], and spray drying also removes volatile metabolites like ethanol [82]. Storage of dried postbiotics over time under different conditions can affect the stability of postbiotic metabolites [83].

Consequently, there is a need for improving laboratory technical and industrial processes that can be used to scale up the production of postbiotics for commercial purposes at cost-effective levels. Dunand et al. [84], in their study, found that both laboratory-scale and industrial-scale production of cell-free postbiotics from milk fermented with *Lactobacilli* strains was comparable in terms of bio-functionality against *Salmonella* infection in a mouse model. There are only a limited number of studies that investigate the scaling of postbiotic production, and it is understandable that constraints could occur at industrial levels due to changes in processing conditions or scaling that can lead to variations in the stability and bioactivity of the postbiotics. More research is required to improve extraction protocols and optimize media and culture conditions for feasibility in the production of postbiotics.

## 5. Characterization of Postbiotics

Several methods have been established and used for the qualitative and/or quantitative examination of postbiotics and a suitable approach is often chosen based on the analytical objectives and the sort of characterization required [8]. The selection of techniques and analytical tools for the identification and characterization of postbiotics depends on the analytical goals and qualitative and/or quantitative characteristics of microbial metabolite complexes, different analytical techniques are currently being used to identify postbiotic metabolites [5]. Very recently, Moradi et al. [61] discussed a variety of analytical methods used to look at the chemical makeup of postbiotics. Gas chromatography (GC), high-performance liquid chromatography (HPLC), thin-layer chromatography (TLC), and spectroscopic methods are the key techniques employed for a qualitative and/or quantitative analysis of postbiotics. GC is the most frequently used analytical tool for the quantitative and qualitative analysis of postbiotics [60,85,86]. Previously, Ricci et al. [87] employed the headspace solid-phase microextraction GC-MS technique to characterize the volatile profile of postbiotics from *Lactobacillus casei*, and they found sixty-two compounds. Similarly, GC was used to study the short-chain fatty acid content of postbiotics of four different bacterial strains [87]. HPLC is also a most-frequently-used analytical tool for the quantitative and qualitative analysis of postbiotics [87,88]. Due to its high efficiency, resolution, sensitivity, and accuracy, as well as its minimal usage of solvents, ultra-performance liquid chromatography represents superior separation and identification capabilities for postbiotics [89,90,91,92]. Sharma et al. [93] and Wang et al. [94] have used TLC and confirmed the presence of various compounds in postbiotics. Indeed, colorimetric methods have been studied to quantify metabolites in the postbiotics of LAB [95]. Lin and Pan [89] and Arasu et al. [96] have explored it using nuclear magnetic resonance spectroscopy as a potential tool to learn the interactions of biological metabolites of postbiotics. In another work, Shafipour Yordshahi et al. [78] examined postbiotics from *Lactobacillus plantarum* using the Fourier transform infrared spectroscopy technique. Indeed, in postbiotics, the analytical methods utilized must be the most suitable for locating the desired metabolite or discovering novel metabolites. A method that enables the identification of a broad range of metabolites yields, more desirable findings and the characterization technique must be selected accordingly.

## 6. Biological Activities of Postbiotics

### 6.1. In Vitro Studies of Bioactivities

Postbiotic therapies are not known to have any adverse effects like inflammation, etc., during clinical and experimental procedures. Indulged with its safer dosages and known chemical structures it can be proposed as a safe alternative to probiotics engrossment. Owing to their exclusive competence in the prevention and treatment of certain diseases and improvement of health status, there is an increased use of these postbiotics in veterinary, medical, and food applications. Among the different properties exhibited by postbiotics, the anticancerous activity of these metabolites is promising and of medical significance. Being a multifactor disease encompassing the irregular or uncontrolled growth of cells, the traditional methods of treatment results in resistance to chemotherapy, the toxicity of the system, and even the reoccurrence of cancer growth [97]. Mainly the involvement of postbiotics in cancer treatment is associated with their capacity to connect with the host immune cells, activating certain signaling routes, which results in an upsurge of inherent immune system reaction and reduction in inflammation [98]. In vitro analysis of exopolysaccharide metabolites from the postbiotic strain of *L*. *plantarum* depicts the prospect of the convention of these metabolites in functional food supplements and their use as antitumor drugs [99]. Explorative studies on the effects of metabolites showed that there is dosage and period dependency in results of the inhibition of liver cancer, gastric cancer, and colon cancer cells. Moderate antitumor activity was observed in the case of liver cancer cells and a significant inhibition ratio of around 61% and 88.34% against gastric and colon cancer cells. Similarly, metabolites of six postbiotic strains of *L*. *plantarum* also exhibited strain, time, and dosage-dependent cytotoxicity against different cancerous cell lines with an inhibition concentration of about 50% [100]. There was no validated toxicity of normal human breast cells; red blood cells of humans, dogs, rabbits, chickens; and of mice splenocytes. Further clinical studies are required to optimize the delivery approaches, design suitable carriers, and assess the potential for postbiotics following oral treatment as well as the underlying processes of cell death for their uses in vivo.

Additionally, in vitro revisions of postbiotic-constituent mechanisms against the oxidative components is evidently an unresearched area which entails more consideration and research. Chang et al. [101] revise the significance of *Lactiplantibacillus plantarum* strains as a potential antioxidative source. The author draws a positive connection between the organic acid production and intracellular components like pyrrole derivatives among postbiotics metabolites and accomplished oxidative inhibitory activity. In vitro evaluations of multifunctional properties of postbiotic strains discloses the antioxidant activities of intracellular contents and cellular fractions of *Lactobacillus casei* and *Bacillus coagulans* strains [72]. Studies depicted a cellular antioxidant activity (CAA units expressed as quercetin equivalents) that ranged from 22.7–42.9 CAA units in the case of *Lactobacillus casei* and 43.1–56.9 CAA units in the case of *Bacillus coagulans*. Reports on exploratory studies on the effectiveness and anti-microbial potential of postbiotic strains were confirmed by several authors. The potential for postbiotics obtained from three different strains of *Lactobacillus spp.* in preventing the colonization of pathogenic organisms was reported by Moradi et al. [81]. Residual antimicrobial activity of over 50% was observed in the case of all three strains of LAB species with significant reductions in the case of the biofilm formation of *Listeria monocytogenes*. The author correlates the concept of controlled pathogenic growth to the postbiotic secretion of different substances like bacteriocins, hydrogen peroxide, organic acids, etc., which hinder microbial growth. Prevention of microbial growth is dependent on certain factors including the type of postbiotic substances involved, contact time, the process of the carnage of prebiotic strain, and concentration. Alongside the reported variance in the inhibitory activity among different species or strains, there is a possibility of difference in repressive nature according to the killing method of prebiotic strain. The inhibitory effect of *Acetobacter pasteurianus* and *Lactobacillus crustorum* strains were found to be affected by the killing method while examining the effectiveness in the prevention of growth of *Streptococcus mutans* and *Escherichia coli* [102]. Among the two different strains, *S*. *mutans* were found to more sensitive to postbiotics than *E*. *coli*, exhibiting a total of 70% diminution in viability. Among the different methods of carnage involved, the heat-killed bacteria to some extent was ineffective against the populations of *E*. *coli*. Formaldehyde killed method was found to be effective against the populations of *E*. *coli* and *S*. *mutans*, with more than a 50% decrease in viability. With little knowledge of the disparity in mechanisms concerning varied results, there should be thorough studies on various factors shaping the efficacy of the process. The antiviral potential of these postbiotics is well thought out as a supplementary benefit to their antibacterial properties. The strains of *Lactobacillus amylovorus*, *Lactobacillus plantarum*, and *Enterococcus hirae* exhibited good antiviral properties against the recovered enterovirus isolates [103]. The antiviral effects of these strains are proposed to different mechanisms including the production of hydrogen peroxide and organic acids and competitive exclusion. Postbiotic metabolites like amino acids, bacteriocins, SCFAs, peptidoglycan-derived muropeptide, vitamins, etc., promote and enhance the anti-inflammatory functions in the human gut. Clinical trials on the anti-inflammatory potential of postbiotics of the *Bifidobacterium longum* strain showed that it was effective in controlling the acute inflammatory response and gut disruption by activating the pathways related to distinctive immune response [104]. The study explains that the potential of the heat-treated probiotic strains was in agreement with the gut-barrier protective capacity and anti-inflammatory potential of the live strain. As with every functional activity of postbiotics, the effectiveness of metabolites in controlling the inflammatory condition is dependent on several factors, especially the type of postbiotic strain. Lin et al. [105] evaluated the dependency of metabolite efficiency with the strains involved. Multi-strain postbiotics was exhibiting a superior anti-inflammatory potential compared to single-strain postbiotics and efficiency of postbiotic metabolites is closely dependent on the choice of strains integrated together. The results explain that the multi-strain probiotics named as probiotic extracts of four strains—number 1, PE0401 which is an amalgamation of four probiotic strains including *Lactobacillus salivarius*, *Lactobacillus plantarum*, *Lactobacillus acidophilus*, and *Bifidobacterium longum* in 1:1:1:1 ratio was effective against all the other mixed forms of the metabolites.

### 6.2. In Vivo Studies of Bioactivities

Studies on postbiotics have a known level of adherence to the characteristics of prebiotic pattern and environment. However, it does not degenerate the efficiency of postbiotics in fostering their antimicrobial, antidiabetic, antiproliferative, antioxidant and anticancer roles in living organisms. An advantage of postbiotics over other chemical preservatives and antibiotics is their effectiveness against pathogenic bacteria solving the possibility of reducing food spoilage issues. The antimicrobial properties of postbiotics are dependent on factors like the type of the target microorganism, postbiotic concentration, and the nature of the source prebiotics involved. Probiotic stimulation achieved due to the presence of *Lactobacillus* spp. in mice helped in reducing the infectivity and total activity of *E*. *coli* preventing intestinal inflammation in a broader perspective [106]. The incidence of postbiotics has been found to elevate the level of cytokines arbitrated habitually by pathogenic recognition receptors inducing immune homeostasis against the targeted organism. Correspondingly, the efficacy of *Lactobacillus* spp. against *L*. *monocytogenes* in both in vivo and in vitro studies was reported by Nakamura et al. [11]. Inhibition of activity of around 90% was made possible by the postbiotic orientation in live tissues. Theories on the effectiveness of these populations revolve either around artificially reduced nutrient availability or pH decreases indicating the presence of LAB bacteria. Additionally, the antiviral activity of postbiotics is a significant diversion that requires deeper thought. Postbiotics derived from populations of *Lactobacillus plantarum* were effective against SARS-CoV-2 infection by modifying the immune system and attacking the virus, as discussed by Anwar et al. [107]. Plant-derived probiotics were helpful in controlling the pathogenicity of the Coronavirus 2019 (COVID-19) by affixing itself with the spike glycoprotein which otherwise shows a possibility of binding with angiotensin 2 converting enzyme (ACE2) causing infection. Table 1 summarizes the beneficial effects of postbiotics observed in in vivo studies.

Defensive mechanisms of postbiotics constituents, especially against the oxidative properties of different elements, were studied and reported by several authors. Dietary postbiotics of *L*. *plantarum* strain reduced the serum lipid peroxidation and boosted the serum and ruminal fluid antioxidant activity with augmented hepatic antioxidant enzymes [108]. Postbiotics of the indicated strain augment the antioxidant activities by exhibiting a high resistance to hydrogen peroxide and elevated rates of scavenging activity against superoxide, hydroxyl, and DDPH free radicals beholden to the presence of metabolites including EPS, lipoteichoic acid, and cell-surface proteins [109]. The incorporation of postbiotics as an oral supplement in broilers positively influenced the antioxidant properties enhancing the activity of enzymes like glutathione peroxidase and decreasing heat-stress markers. The author accounts for the likelihood of the introduction of postbiotic strains of *Lactiplantibacillus plantarum* as an antibiotic substitute and natural antioxidant source in feeding patterns [110]. In vivo studies of postbiotic metabolites require a better understanding of the delivery and reaction mechanisms, signaling pathways, and of the direct effects of constituents on the living cells. With limited information on these scales, in vivo experimental research on the anticancer potential of postbiotics is still in its infancy. Chen et al. [111] elucidate the efficacy of metabolites of *Clostridium butyricum* in modulating and inhibiting intestinal tumor development in mice. The likelihood of the suppression of tumor growth is retracted to the efficiency of butyrate-producing bacteria in Wnt signal modulation and gut microbiota. More experiential learning are needed to optimize and extend the application ranges of this postbiotic potential. Postbiotics are known to enhance the immune system of beings by varying and strengthening the microflora of the intestinal tract and thereby preventing inflammatory issues in the body Rad et al. [112]. LAB are known for modulating the immune responses in normal conditions which has been evaluated by Menard et al. [113] in mice. The study depicts the clear correlation between the anti-inflammatory effect of postbiotics of *Bifidobacterium breve* and *Streptococcus thermophilus* strains in an inflammatory context and their immune stimulatory effects. Strains of *Lactobacillus bulgaricus* and *Streptococcus thermophilus* are found to yield health effects in mice by preventing the advance of colitis a chronic inflammatory disease [114].

**Table 1:** Beneficial effects of postbiotics observed in in vivo studies.

Biological Activity	Strain Used	Observation	Reference
Antimicrobial properties	*Lactobacillus* spp.	Diminution in infectivity and total activity of *E. coli* preventing the intestinal inflammation in mice	[106]
Antiviral properties	*Leuconostoc mesenteroides*	Metabolites increased IFN-c cytokine production and modified the immunity of broilers against influenza virus (H9N2)	[115]
Antioxidant properties	*L. plantarum*	Reduction in serum lipid peroxidation in mice, boosting serum and ruminal fluid antioxidant activity	[108]
Anti-inflammatory properties	*Lactobacillus bulgaricus* and *Streptococcus thermophilus*	Efficacious in controlling the advance of colitis in mice	[114]
Antioxidant properties	*Lactobacillus* *plantarum*	Aided in enhancing the total activity of enzymes by oral supplementation in broilers	[116]
Anticancer properties	*Lactobacillus casei* and *Lactobacillus paracasei*	Reduce the rate of proliferation and apoptosis	[117]
Antimicrobial properties	*Lactobacillus* spp.	Inhibition of activity around 90% of *L. monocytogenes* in both in vivo and in vitro studies	[70]
Antiviral properties	*Lactobacillus plantarum*	Operative against SARS-CoV-2 infection by altering the immune system	[107]
Antitumor properties	*Clostridium butyricum*	Suppression of tumor growth due to oral supplementation in mice	[111]
Anti-inflammatory properties	*Bifidobacterium breve* and *Streptococcus thermophilus*	Successful in modulating the immune stimulatory effects in mice	[113]
Antioxidant properties	*Lactiplantibacillus plantarum*	Increased the activity of enzymes like glutathione peroxidase when included in dietary pattern of broilers	[110]

### 6.3. Infection Prevention

Postbiotics are defined as the preparation of inactive microorganisms and/or their parts that benefits the host’s health [118]. A novel method for obtaining probiotic bacteria’s positive effects without their potential drawbacks is the use of postbiotics. Despite the rarity of probiotic-related infections and the ongoing debate over whether probiotics could express virulence factors or pass antibiotic-resistant genes to pathogenic bacteria [119,120], using postbiotics might avoid those problems because the bacteria are rendered inactive by heat, high pressure, sonication, or ionizing radiation [121]. Numerous studies claim that postbiotics can enhance physiological functions [122] and prevent and treat diseases including gastroenteritis, respiratory tract infections, and enteric infections as well as conditions like gut barrier dysfunction [26,84,123,124]. Some postbiotics can compete for receptors needed by some pathogenic bacteria, seal the intestinal barrier, change the expression of host genes, or modify the local environment to have direct antimicrobial effects [40].

#### 6.3.1. Gastroenteritis

For the treatment and prevention of gastrointestinal illnesses, prebiotics and, more recently, postbiotics are being employed. Children and young animals, such as calves and piglets, are susceptible to diarrhea, vomiting, and fever brought on by the rotavirus (RV), a nonenveloped virus of the family Reoviridae. A recent study [122] investigated the results of daily supplementation with a formulation based on a postbiotic and prebiotic combination, similar to what is found in breast milk, in a model of RV infection in suckling rats. The authors concluded that Lactofidus^™^, short-chain galactooligosaccharides (scGOS)/long-chain fructooligosachharide (lcFOS) and their combination prevented, and reduced diarrhea brought on by RV in suckling rats. The immunoglobulin profile, intestinal gene expression, and gut flora all changed in response to supplements. Both scGOS/lcFOS and Lactofidus^™^ appear to treat diarrhea in a distinct way. The postbiotic decreased pH in feces and increased toll-like receptors (TLR) expression. The authors concluded that most individually observed benefits were maintained when scGOS/lcFOS and Lactofidus^™^ were combined, and certain effects (such as an increase in white blood cell and lymphocyte recruitment and an elevation of TLR7 and TLR9 gene expression) were even noted. Similarly, in another randomized control trial (RCT), the effectiveness of daily feedings of a fermented formula containing non-live *Bifidobacterium breve* C50 and *Streptococcus thermophilus* 065 for five months was examined in 913 otherwise healthy French infants (ages 4 and 6 months). Between the intervention group and the placebo group, there was no difference in the number of diarrheal episodes (*n* = 913, RR 1.01, 95% CI 0.98 to 1.04) [125]. Additionally, the effect of giving heat-killed *Lactobacillus paracasei* CBA L74 for 90 days on avoiding diarrhea in 537 healthy Italian kids between the ages of 12 and 48 months were also studied [126,127]. The authors reported a significant reduction in diarrheal episodes in the heat-killed *Lactobacillus paracasei* group as compared to the placebo group (2 RCTs, *n* = 537, RR 0.51, 95% CI 0.37 to 0.71).

#### 6.3.2. Respiratory Tract Infections

For young children attending daycare or preschool, respiratory tract infections are a serious issue, especially during the winter months. The immune system, as well as the respiratory and digestive systems, are generally immature, which facilitates these illnesses, known as common infectious diseases (CIDs). Acute upper respiratory tract infections (URTIs), which include the common cold, acute sinusitis, acute pharyngitis, acute laryngotracheobronchitis (croup), acute epiglottitis (supraglottitis), acute rhinosinusitis, and acute otitis media (AOM), are a significant cause of morbidity, particularly in children and the elderly.

The impact of postbiotics on respiratory tract infections has also been studied [126,127]. The effectiveness of giving 537 healthy Italian children the heat-killed *Lactobacillus paracasei* CBA L74 for 90 days of avoiding respiratory tract infections was examined in two randomized control trials (RCT). The combined analysis of these two studies showed that the use of postbiotics decreased the incidence of pharyngitis (RR 0.31, 95% CI 0.12 to 0.83; NNT 2.4, 95% CI 1.9 to 3.1), laryngitis (RR 0.44, 95% CI 0.29 to 0.67; NNT 3.6, 95% CI 2.5 to 6.5), and tracheitis (RR 0.65, 95% CI 0.50 to 0.86; NNT 8.05, 95% CI 4.5 to 32.4). Between the postbiotic and placebo groups, there was no significant difference in the risk of rhinitis (RR 0.77, 95% CI 0.57 to 1.03; I^2^ = 0%) or otitis media (RR 0.36, 95% CI 0.13 to 1.02; I^2^ = 69%) [126,127]. The authors [126] observed a significant increase in the production of innate and acquired immunity peptides (an immunostimulatory impact). Innate immunity peptides serve as endogenous antimicrobial agents and protect the body from a wide variety of pathogens. They are produced by epithelial cells, Paneth cells, neutrophils, and macrophages (bacteria, fungi, protozoa, and viruses). These peptides have also been demonstrated to control the function of macrophages, dendritic cells, T cells, monocytes, and neutrophils in addition to their antibacterial properties. Additionally, the fermented food item had a favorable impact on the generation of sIgA, a crucial element of acquired immunity. In addition to providing the first line of defense against pathogens in the mucosae, sIgAs also control the composition of the gut microbiota, facilitating communication between gut commensal bacteria and the host immune system [126].

#### 6.3.3. Allergic Diseases

Due to their ability to balance Th1/Th2-mediated immune responses and facilitate immune system maturation, postbiotics are thought to be a potential therapeutic approach for allergic disorders. The use of postbiotics in preventing asthma and wheezing exacerbations in children is, in fact, supported by the data currently available [128,129]. Additionally, the quantity of butyrate-producing bacteria in the colon was negatively correlated with the severity of atopic dermatitis symptoms [130], and oral bacterial lysate (BL) intake was linked to better atopic dermatitis treatment outcomes in children [131]. Additionally, postbiotics might help people with food allergies. An extensive butyrate-producing bacterial microbiota was linked to a quicker remission of cow’s milk allergy, according to a clinical investigation involving more than 200 kids.

#### 6.3.4. Enteric Infection

The mucosa-associated immune system’s most prevalent defense, IgA, serves as an immunological excluder of harmful bacteria and viruses as its primary function [132]. Several studies have reported a reduction in the severity of enteric infections brought on by pathogens like *Salmonella enteritidis* serovar *Typhimurium* or *Escherichia coli*. The possible explanation for this reduction can be attributed to increasing the levels of gut IgA in the small and large intestine lamina propria of mice as a result of postbiotic administration [133].

In another study, a mouse model exposed to an acetate-rich diet showed a considerable improvement in resistance to enterohaemorrhagic *E*. *coli* O157:H7 infection. This occurrence is probably caused by the intestinal barrier’s ability to seal off harmful poisons, preventing them from reaching the bloodstream [134]. Another study demonstrated that the nonbacterial fraction (NBF) of milk fermented with *Lactobacillus helveticus* R839 has a protective effect against *Salmonella Typhimurium* infection [135]. The authors concluded that animals given NBF on day 7 post-challenge had lower liver colonization levels, greater luminal contents of specific anti-Salmonella S-IgA, and fewer MIP-1a+ cells in the lamina propria. Similarly, in another study, the ability of the postbiotics created by pH-controlled fermentation to promote secretory IgA synthesis in feces and shield mice from a Salmonella infection was assessed [84]. In comparison to control animals, a substantial rise in secretory IgA was found in the feces of mice fed with the laboratory-produced postbiotic (F36) for 14 days [84].

#### 6.3.5. Other Clinical Applications

Some researchers also indicated that postbiotics may also be helpful in the prevention and treatment of SARS-CoV-2 infection since the gut microbiome’s structure and metabolic activity may be connected to the appearance of biomarkers predicting the severity of the severe Coronavirus disease 2019 (COVID-19) course [136]. Modulation of the gut microbiota with probiotics appears to have an impact on the COVID-19 inflammatory storm and directly inhibits SARS-CoV-2 proliferation [137]. So, in order to protect susceptible persons from contracting SARS-CoV-2, GM remodulation using postbiotics can be extremely important. Additionally, GM remodulation can alter COVID-19’s biology to take on a milder form [138]. Postbiotics could directly suppress SASR-COV-2 replication similarly to probiotics.

Similarly, another study suggested that bacterial lipoteichoic acid (LTA) may be effective in treating a variety of skin infections. For some bacterial and viral infections, *Lactobacillus* and *Bifidobacteria* genus bacteria trigger the skin’s mast cell response [139]. Gram-positive bacteria’s cell walls contain LTA, which can spontaneously release into the environment. By enhancing non-specific defense mechanisms, LTA applied topically triggers the release of anti-infectious peptides like human defensin and cathelicidin [140]. Non-pathogenic bacteria that cause opportunistic infections become pathogenic when the body’s defensive mechanism is compromised [141]. *Candida* spp. is associated with opportunistic yeast infections and can cause vaginitis, oral candidiasis, cutaneous candidiasis, and candidemia [142]. A study by Rossoni et al. [142] investigated the antifungal efficacy of crude extract (LPCE) and fraction 1 (LPF1), which were obtained from *Lactobacillus paracasei* 28.4 supernatant, utilizing in vitro and in vivo models of probiotic *Lactobacillus paracasei* 28.4 cells [142]. According to the study, *Lactobacillus paracasei* 28.4 cells and postbiotic components (LPCE and LPF1) have antifungal action against *Candida auris* planktonic cells, biofilms, and persister cells.

The application of postbiotics for Necrotizing Enterocolitis (NEC) has also received some attention. Necrotizing enterocolitis (NEC) is one of the major causes of infant morbidity and mortality; hence, there is an urgent need for prophylactic medicines that are both efficient and secure. Probiotics are now the most potent treatment on the horizon for this deadly illness, according to recent evidence from therapeutic studies that suggest they are beneficial in reducing NEC in preterm newborns. However, issues with safety and dosage have restricted the use of probiotics in preterm newborns in typical clinical settings. Although research confirming postbiotics’ clinical usefulness in preventing NEC is limited, they may be possible substitutes for or complementary therapy to the injection of live microorganisms. Important bacterial structures could still have biological action in the host even after being heat destroyed by microorganisms. In a murine model of immature intestines, a study [143] found that heat-killed *Lactobacillus rhamnosus* GG (LGG) has equal therapeutic efficacy to live probiotics in speeding intestinal barrier maturity. It was important to notice that whereas giving live LGG in larger doses increased mortality in immature neonatal mice, giving heat-killed LGG did not. The authors [144] emphasized the need for further clinical trials to conclude postbiotic’s effect on the clinical outcomes of prematurity, such as NEC, sepsis, or death.

There has also been considerable interest in the use of postbiotics in place of antibiotics in agricultural animals. Finding alternatives to produce antimicrobial effects against common pathogens in large-scale farms have become more popular as a result of the banning of antibiotics in several countries. A summary of some of the postbiotic applications in bovine farming is listed in Table 2.

The mechanisms of action of postbiotics are yet to be fully understood. It has been suggested that inactivated probiotics or their constituent parts may modify the host’s immunological response. The bacterial pellicle, capsule, or cell-wall constituents [152] such as peptidoglycans, liposaccharides [153], and S-layer proteins [154] can all stimulate the immune system. A number of pattern recognition receptors (PRRs) are present in the early host response. These PRRs are proteins that are primarily expressed by natural immune system cells and that are able to identify chemicals peculiar to microbes known as pathogen-associated molecular patterns (PAMPs). The nucleotide-binding oligomerization domain (NOD)-like receptors (NLRs) and toll-like receptors (TLRs) have been postulated as two types of PRRs that play major roles in the control of the host’s innate immune response. The various TLR subtypes can bind to a variety of microbial components, including bacterial nucleic acids (DNA or RNA), peptides (such as flabellin), lipoproteins, lipoteichoic acids, and peptidoglycans [155]. The innate and acquired (adaptive) immune responses [155] are both mediated by the NLRs, PRRs that are comparable to TLRs. The NLRs can detect a variety of ligands from microbial pathogens, including flagellin, peptidoglycans, and viral RNA [156]. It has also been suggested that certain cytokines, such as interferons, may cause the NLRs to respond [157]. In this way, NLRs might help T and B cells respond to postbiotic cues by activating them. Reactive oxygen species (ROS) and IL-2 production may be decreased while the IL-4, IL-6, and IL-10 production may be increased as a result of postbiotic product components like lipoteichoic acid [158]. Overall, in areas where probiotic administration is difficult or impossible, postbiotics may be an attractive alternative since they eliminate the need to maintain the bacteria’s viability while keeping the benefits of lowering the incidence of common infectious disorders.

## 7. Potential Applications in Food and Pharmaceutical Sector

Functional foods that contain probiotics, prebiotics, and postbiotics have drawn a lot of interest from researchers, manufacturers, and consumers in recent years. A significant amount of postbiotic research is currently focused on not only accurately defining their mechanisms of action but also creating novel functional food and preventative medication formulations for improving host health [159]. There are a wide variety of food products with bioactive substances like probiotics, dairy, and non-dairy products already on the market [57] to suit the nutritional needs of customers with diverse dietary preferences, such as people who are allergic to milk proteins, lactose intolerant, and vegetarians [159]. Since postbiotics are stable across wide temperatures and pH ranges, it is possible to add foods and ingredients before thermal processing without compromising their functionality. This could give producers some technical and financial advantages [159]. Postbiotics can be used in delivery systems like functional foods and/or pharmaceutical products because their adequate amount can be controlled during production and storage conditions, where survival is not the main determining factor.

### 7.1. Potential Role of Postbiotics in the Food Industry

Postbiotics can be composed of bacterial lysates with cell surface proteins, bacterial enzymes, peptides, metabolites (produced by bacteria such as teichoic acids), neuropeptides (derived from peptidoglycans, polysaccharides), and lower organic acids like lactic acid [10,158,159]. Fermentation is the most prevalent postbiotic source in the food industry. The presence of postbiotics can be found naturally in several milk-based and other products like kefir, kombucha, yogurt, and pickled vegetables [57]. Table 3 provides a summary of the applications and benefits of postbiotics in food products. The producer strains mostly include strains of *Lactobacillus* and *Bifidobacterium* but may also include *Streptococcus*, *Akkermansia muciniphila*, *Eubacaterium hallii*, *Faecalibacterium*, *Saccharomyces boulardii* and can be used to extract the postbiotics in situ [10,56]. EPS are extracellular biopolymers that microbes produce or secrete during the course of their growth. They differ greatly in both the amount of branching they exhibit—from linear molecules to highly branched molecules and the monosaccharide content of these molecules [160]. EPS produced by LAB, such as *Lactobacillus rhamnosus*, plays a significant role in dairy products and can enhance the physicochemical and sensory qualities of food-based products [10]. Similarly, another study found that the postbiotic supernatant from *Lactobacillus plantarum* YML007 can be a possible bio-preservative, extending the shelf life of soybeans by up to two months [161]. Additionally, a number of bacteriocins have been isolated, characterized, and may have potential industrial uses. The microbial strain and culture conditions will determine their isolation and characterization. They must first be biologically inactive before being altered to become active [162]. Nisin, which is known as a preservative in several foods (infant formula, canned soups, dairy products), can be produced by *Lactococcus lactis* subsp. *Lactis* [163]. Several studies have also focused on the use of enzymes instead of probiotic bacteria to achieve specific effects. Postbiotic enzymes such as purified phytases from *Bifidobacterium pseudocatenulatum* and *Bifidobacterium longum* spp. *infantis*, for example, decreased the amount of phytates in cereal combinations and elevated myoinositol triphosphate levels [164]. Vitamin enrichment in food products has also been reported by several authors [165,166]. Increasing vitamin B as a result of fermentation is a very common strategy applied in cereal grains. Cereal grains are rich in vitamin B. However, during milling or heat processing, these vitamins are lost. Cereal fermentation and LAB pre-treatment increase the amount of bacteria that can produce the vitamins B1, B2, B3, B9, B11, and B12. In vitro levels of total lysine, protein fractions, sugars, soluble dietary fiber, and bioavailability of Ca, Fe, and Zn were all dramatically increased by the LAB fermentation of cereals. Additionally, wheat could produce antihypertensive angiotensin I-converting enzyme-inhibitory peptides, γ-aminobutyric acid, and antioxidant peptides by LAB fermentation [167].

Another novel approach to probiotic usage also involves removing some potentially toxic food ingredients during probiotic-induced fermentation in addition to supplementing food with postbiotics. A study by Sarno et al. [168] reported that the amount of toxic gliadin peptides is reduced in celiac patients by fermentation with the probiotic bacteria *Lactobacillus paracasei* CBA L74 [168]. With the aforementioned in mind, the use of foods as a delivery vehicle for postbiotics appears to be a field with many prospects but also some challenges.

**Table 3:** Application and benefits of postbiotics in food products.

Postbiotic	Food	Benefits	References
Nisin	Dairy products, processed products, soups, sauces	Acts as a preservative	[9]
Polysaccharide extracts from *Lactarius volemus* Fr.	Yoghurts	Improvement in water retention and reduction in pH	[169]
Supernatant of *Lactobacillus plantarum* YML 007	Soybeans	Increased shelf life until 2 moths	[170]
*Lactobacillus rhamnosus* exopolysaccharide	Cheddar cheese	Improved product performance	[171]
Purified physates from *Bifidobacterium longum* spp. *infantis* and *Bifidobacterium pseudocatenulatum*	Cereal mixtures	Reduced contents of physates and increased level of myo-inositol triphosphate	[172]
*Lactobacillus rhamnosus* S93 enzyme	Cheddar cheese	Higher concentrations of soluble nitrogen in free amino acids and phosphotungstatic acid	[173]
Supernatant of *Lactobacillus sakei* NRRL B-1917	Grilled beef	Reduced counts of *E. coli* and *Listeria monocytogenes*	[174]
Bacteriocin—*Lactobacillus gasseri* LA39 Gassericin	Custard cream	Complete inhibition of four decomposition strains	[175]
Peptides—Released by Casein hydrolysis	Dairy Products	Antihypertensive	[176,177]
Bacteriocin—*Lactobacillus coryniformis* MXJ 32	Food in general	Bactericide for *Staphylococcus aureus* and *Escherichia coli*	[178]
Bacteriocin-like inhibitor substance of *Lactobacillus plantarum* ST16Pa	Chicken breast	Bioconservative against *Enterococcus faecium* for 7 days	[179]
Pirrolo [1,2-a] and pyrazine-1,4-dione from *Lactobacillus salivarius*	Ground beef and whole milk	Biofilm removal of *Listeria monocytogenes*	[81]

### 7.2. Potential Role of Postbiotics in Pharmaceutical or Health Industry

Numerous postbiotic molecules have attracted interest because of their unique properties such as their chemical makeup, prolonged storage stability, and capacity to activate various mechanisms regulating inflammation, obesity, hypertension, cardiovascular disease, cancer, and oxidative stress [160]. Postbiotics provide immunomodulatory activity to the host and may represent a safer alternative when the use of live probiotic bacteria is not indicated. A recent review by [63] highlighted several pharmacodynamic features of postbiotics over probiotics which are (1) Invulnerable and immunocompromised individuals have minimal or no risk of bacterial translocation from the stomach lumen to blood; (2) No possibility of acquiring and transferring genes for antibiotic resistance; (3) The extraction, standardization, transportation, and storage is more natural; (4) Additional positive effects may result from cell lysis-induced viability loss; (5) Improved interactions between the epithelial cells and molecules released from the disrupted cells [63].

Nowadays, industries are attempting to incorporate postbiotics into the pharmaceutical product matrix due to their beneficial role in clinical scenarios. For example, CytoFlora^®^ manufactured by BioRay Inc., Laguna Hills, CA, USA is a well-known postbiotic in the pharmaceutical industry. CytoFlora^®^ is composed of cell walls isolated from bacteria some of which include *Lactobacillus casei*, *Lactobacillus plantarum*, *and Lactobacillus acidophilus* DDS-1. CytoFlora has been reported to have several important positive impacts, including preserving intestinal homeostasis, enhancing intestinal dysbiosis, enhancing immunological response, and alleviating symptoms in autistic individuals [10,57,159]. Del-Immune V^®^ from Pure Research Products, LLC, Boulder, CO, USA is another postbiotic pharmaceutical product. It is typically advised to take them with probiotics for therapeutic purposes. A combination of amino acids, muramyl peptides, and DNA fragments from *Lactobacillus rhamnosus* V make up the Del-Immune V formulation. Studies have shown that Del-Immune V significantly lessens the severity of the gastrointestinal disorder in people with autism spectrum disorder [10,159]. In another example, for the treatment of intestinal active inflammatory diseases, Zakofalk^®^ (a mixture of sodium butyrate and inulin) is frequently advised [159]. A sterile liquid postbiotic medication called Hylak^®^ Forte (Ratiopharm/Merckle, Germany) contains biological metabolites such as organic acids, short-chain fatty acids, and other metabolites. The development of gut-beneficial microbes, regulation of gut environment pH, support for healthy digestion, energy supply for intestinal epithelial cells, control of vitamin K and B balances, and treatment of salmonellosis and intestinal disorders in adults and children with chronic gut disorders are some of the main health effects of Hylak^®^ Forte [159,180].

In addition to the commercial availability of postbiotic products, much attention has been paid to various health benefits that could result in the future production of other drugs. For example, reproductive health and the impact of postbiotics were recently reviewed [4]. They supported the use of postbiotics as a promising tool in a personalized medicine approach for restoring vaginal eubiosis/health. Additionally, it was recommended for both preventative and adjuvant treatment strategies in women with reproductive-related disorders due to their unique features in terms of clinical, technological, and economic aspects [4]. Researchers also reviewed the impact of postbiotics in children younger than 5 years old and recommended heat-killed *Lactobacillus acidophilus* LB for the management of acute diarrhea based on limited evidence. Additionally, the authors also recommended heat-killed *Lactobacillus paracasei* CBA L74 for the prevention of gastrointestinal and respiratory tract infections based on limited evidence and suggested further need for studies to impart further understanding [124]. Similarly, another research group studied the antibacterial mechanism of indole propionic acid, a gut microbiota metabolite, and reported it as an anti-tubercular agent [181,182]. They discovered that indole propionic acid might imitate the physiological allosteric inhibitor of TrpE, limit tryptophan production in *M*. *tuberculosis*, and hence, exhibit antimycobacterial activity after conducting metabolic, chemical rescue, genetic, and biochemical tests [182].

Animal health has also received some attention similar to human health as it offers a promising tool in veterinary research [183,184]. A study reported increased final body weight, total weight gain, and the height of duodenal and ileal villus in broilers when their meals were fortified with postbiotics of *Lactobacillus plantarum* [184]. In another study, supplementing animal meals with *Lactobacillus plantarum* postbiotics increased the length of mucosal villi and strengthened the population of beneficial intestinal microbiota, which in turn improved protein digestibility and growth efficiency, and decreased the incidence of diarrhea [10]. Additionally, postbiotic use changed the intestinal microbiota, increased the number of protective bacteria (such as *Lactobacillus* and *Bifidobacterium*), and improved the health of the test animals. Postbiotics may be helpful as preventative medications, fermented functional foods, and microbial-free food supplements as supplemental therapy for a number of disorders [10].

In summary, there have been numerous postbiotics introduced into the pharmaceutical, veterinary, and food industries as medications, food, and feed, demonstrating an extraordinary aptitude for the prevention and treatment of specific diseases, boosting animal health status, and generating functional foods. Consequently, postbiotics may be a secure substitute and a novel application approach in the pharmaceutical and/or food industries for fostering and developing health advantages, as well as for preventing diseases and achieving therapeutic goals. However, food and drug product’s safety heavily depends on the safety of the ingredients that make up those products. Therefore, additional research is necessary to define novel postbiotics and analyze their stability and safety criteria in the pharmaceutical and food industries. Clinical trials are also required to establish the ideal postbiotic administration frequency and dosage in a delivery method (pharmaceutical formulations and/or functional postbiotic meals).

## 8. Challenges and Safety Regulations

Consumers’ interest in healthy goods has grown in recent years, and the great ability of probiotics to offer favorable health benefits has led to a surge in scientific and commercial interest in microbial administration as a health-promoting technique. There has been significant development in research linked to the relationships between food, microbiota, and host, which has led to microbial administration or modification of human microbiota using innovative therapeutic techniques in recent years. As a result of these recent findings, several terminologies relating to probiotics have been disseminated, but instead of fostering better communication among researchers, government regulators, and the food business, these phrases have instead led to improper usage of the term probiotics [185,186]. Probiotics and prebiotics definitions have recently been reviewed and modified. According to these ideas, a prebiotic is a substrate that is specifically used by host microorganisms, imparting a health advantage, whereas a probiotic consists of live bacteria that, when given to the host in sufficient proportions, confer a health benefit. Many foods and supplements have labels that mention probiotics and prebiotics. Consumer understanding of these phrases is growing, and many countries are labeling them more often for regulatory purposes. In addition, there is mounting evidence that non-viable microbes and the metabolites they can make through fermentation or interaction with dietary components are beneficial for human health [10]. These bioactive substances, which do not fit into the well-known probiotic, prebiotic, or synbiotic categories, have several names such as paraprobiotics [187], parapsychobiotics [188], ghostprobiotics [189], metabiotics [190], tyndallized probiotics [63], and bacterial lysates [191]. So far, the provisional term postbiotics has been the most commonly used. However, as of now, there is still no universally accepted definition of the phrase. Therefore, it is presently of utmost importance to arrive at a common definition of the word “postbiotics.” A definition could also assist in preventing or limiting the term’s misunderstanding, which happens frequently with respect to items that are falsely claimed [186]. The definition and use of postbiotics were reviewed in 2019 by a panel of specialists from the International Scientific Association for Probiotics and Prebiotics (ISAPP) with expertise in food science, nutrition, gastroenterology, microbial physiology, pediatrics, and microbiology [118]. Though a tentative definition of postbiotics was proposed by the panel as “preparation of inanimate microorganisms and/or their components that confers a health benefit on the host”, there is still wide disagreement on the proposed definition among many experts [192]. The need of a proper definition is also significant from a regulatory perspective since it makes it easier for its usage in food and supplements as health promotion tactics to get approved. Additionally, only the fact that postbiotics and paraprobiotics offer biological advantages to the host without the possible risk connected with the administration of live microorganisms already helps to lower legal barriers in the process of legal authorities’ approval.

Currently, there is a bright future for probiotics. The applications of postbiotics in the food domain will benefit a significant portion of the population, as well as people suffering from certain specific medical/disease conditions, where it could play a meaningful therapeutic role. The success of postbiotics, however, will be dependent on a variety of factors, most of which are interrelated. A postbiotic’s capacity to mediate a health effect on an individual may be fueled by a variety of different processes or pathways. A postbiotic could be a complex mixture of components. These pathways may occasionally resemble those identified for probiotics and these mechanisms may operate singly or in combination. Therefore, in order to guarantee that a commercial postbiotic product retains the bioactive properties and other required health-promoting attributes effectively, it is crucial to understand the key molecules responsible for such beneficial effects. Since postbiotics are inanimate, the microbes must also produce these bioactive compounds before inactivation in sufficient quantities to have a positive effect. To determine the ideal target population, the ideal dose, and the ideal form of administration as well as the identification and isolation of the safest and most functional strains still remains as a major research gap which poses a greater challenge for commercialization of postbiotics.

No regulatory authorities, to the best of our knowledge, have developed a postbiotic concept or framework that is particular to foods or dietary supplements that include postbiotics. Some regulatory standards have been established for postbiotic formulations for medical or pharmaceutical purposes [193]. Regarding the evaluation of food safety in Europe, the European Food Safety Authority (EFSA) regulates the standards for food and are updated on a regular basis. Whereas, the European Pharmacopoeia provides defined guidelines that specify maximum permissible amounts of live microorganisms for pharmaceutical preparations and medicinal products [194]. Probiotics, prebiotics, synbiotics, and postbiotics are not specifically covered by any regulations in the European Union; however, numerous postbiotics have been advertised or licensed as immune-stimulating substances [195]. In 2011, Argentina’s food code included Articles 1389 and 1390, which, respectively, refer to probiotics and prebiotics. Even though a multinational company released an infant formula in 2019 that was labelled as containing “postbiotics,” the topic of postbiotics has not yet been covered [118]. The Food and Drug Administration (FDA) in the USA has not spoken particularly on postbiotics. The FDA will likely address postbiotics based on the rules that apply to the individual regulatory category chosen for a product in development because postbiotics can be produced under many regulatory categories. The intended usage, safety, and efficacy of the product must all fulfil the requirements for the relevant regulatory category.

Postbiotics should be safer than probiotics since the bacteria they contain have lost their ability to multiply and hence cannot generate bacteremia or fungaemia, which are hazards linked with probiotic therapy [196]. As postbiotic foods are a relatively new concept, there are no clinical or epidemiological reports of health hazards associated with its consumption, to the best of our knowledge. However, postbiotics cannot be assumed to be safe purely based on the progenitor microorganism’s safety profile. Lipopolysaccharides from Gram-negative bacteria, for instance, can cause sepsis and toxic shock, which is typically lodged in the outer membrane of active bacteria and is released after the cell lysis [197]. Therefore, regulatory advice for postbiotics must be anticipated based on the potential hazards and safety considerations. In this context, a systematic analysis of probiotics in seven randomized controlled clinical studies including a total of 1740 children was examined by Malagon-Rojas et al. [124], where the study looked at the effectiveness of postbiotics in the treatment and prevention of common infectious infections in children under the age of five. The study also looked for some of the possible negative effects of postbiotics and three clinical trials have particularly examined the negative effects of postbiotics in research and reported severe dehydration, higher rate of vomiting and abdominal distention, and pediatric D-lactic acidosis [7,198,199]. A theoretical possibility of developing subclinical accumulation of D-lactate in infants fed with postbiotic acidified and fermented infant formula was also suggested by Łukasik et al. [198]. Moreover, there are currently very few studies that have documented the adverse side effects of postbiotics. Therefore, extensive research both on the laboratory scale and in animal models along with clinical trials is necessary to improve the understanding of the concerning issues related to postbiotic safety. Furthermore, interventional research, randomized clinical trials, and metabolomic studies are required to identify the appropriate dose and optimal consumption frequency, as well as to evaluate the health claims of postbiotics.

## 9. Conclusions and Future Directions

Postbiotic refer to non-viable microorganisms and bacterial-free extracts that may benefit the host by enhancing the bioactivities of probiotics. Eating a diet that is high in probiotic and prebiotic foods could ensure that the gut has an adequate level of these essential compounds as postbiotics are produced when probiotics feed on prebiotics. Postbiotics encompass cell wall components and metabolites produced by live bacteria which have beneficial activities in the host. The bioactivities of postbiotics include anti-cancer, anti-obesogenic, anti-inflammatory, antiproliferative, hypocholesterolemic, antioxidant, and immunomodulatory effects. In the future, clinical investigations are likely to concentrate on their composition in addition to their bioactivity as more information on postbiotics becomes available. Postbiotics are effective, according to the scientific evidence discussed in this review, although the degree of efficacy has not yet been clearly understood. Hence, further research is deemed essential to establish the clinical effectiveness and the prophylactic effects of postbiotics. Further investigations on postbiotics will yield new findings and several beneficial effects to increase their use in the field of food and pharmaceutical industries. Additional studies should be conducted to assess the variables, such as eating patterns, that may affect the usage of these beneficial compounds in functional foods. To further comprehend their mechanisms of action in the host, additional research should be conducted. In addition, the widespread use of postbiotics in food and pharmaceutical industries will raise the need for them to be produced under proper industrial conditions and under rigorous adherence to quality control standards. These efforts will aid in the production of secure, natural clean label products that are also devoid of any residues, protecting human health. Therefore, to attain the added health benefits, the qualities of new postbiotic components must be comprehensively evaluated. To ensure that the food and pharmaceutical sector is profitable and sustainable, the longtime health benefits of using these supplements must also be considered. Thus, postbiotics are constantly evolving and future research directions should emphasize establishing the relation between the health impacts of postbiotics and their specific mechanisms.

## Figures and Tables

**Table 2:** Overview of the application of postbiotics and their beneficial effects in bovine farming.

Postbiotic	Beneficial Effects	References
Nisin	Improved body weight gain, feed conversion ratio, and feed intake indexes in chickens	[144,145]
*Lactobacillus gasseri*, *Limosilactobacillus* *reuteri*, and *Ligilactobacillus* *salivarius*	Bactericidal activity against *Escherichia coli* O157:H7, *Mycobacterium avium* ssp. *paratuberculosis*, and *Salmonella* species	[146]
Bovicin HC5	Bactericidal effect against *L. monocytogenes*, *Salmonella Typhimurium*, and some species of *Clostridium* and *Bacillus*	[147,148]
*Lactobacillus lactis* LL11 and SL153	Bactericidal effect against the most common occurring mastitis pathogen	[149]
Nisin derivatives	Antibacterial activity against *S. aureus*	[150]
Bacteriocin from *Lactobacillus lactis* CJNU 3001	Inhibition of the growth of *S. aureus* KCTC 3881	[151]

## Data Availability

The data presented in this study are available on request from the corresponding author.
